# Identification of Kinases and Phosphatases That Regulate ATG4B Activity by siRNA and Small Molecule Screening in Cells

**DOI:** 10.3389/fcell.2018.00148

**Published:** 2018-11-01

**Authors:** Niccolo Pengo, Krisna Prak, Joana R. Costa, Christin Luft, Alexander Agrotis, Jamie Freeman, Christina A. Gewinner, A. W. Edith Chan, David L. Selwood, Janos Kriston-Vizi, Robin Ketteler

**Affiliations:** ^1^MRC Laboratory for Molecular Cell Biology, University College London, London, United Kingdom; ^2^UCL Cancer Institute, University College London, London, United Kingdom; ^3^Wolfson Institute for Biomedical Research, University College London, London, United Kingdom

**Keywords:** ATG4B, siRNA, small molecule, kinase, phosphatase, cDNA, screen, AKT2

## Abstract

Autophagy protease ATG4B is a key regulator of the LC3/GABARAP conjugation system required for autophagosome formation, maturation and closure. Members of the ATG4 and the LC3/GABARAP family have been implicated in various diseases including cancer, and targeting the ATG4B protease has been suggested as a potential therapeutic anti-cancer strategy. Recently, it has been demonstrated that ATG4B is regulated by multiple post-translational modifications, including phosphorylation and de-phosphorylation. In order to identify regulators of ATG4B activity, we optimized a cell-based luciferase assay based on ATG4B-dependent release of Gaussia luciferase. We applied this assay in a proof-of-concept small molecule compound screen and identified activating compounds that increase cellular ATG4B activity. Next, we performed a high-throughput screen to identify kinases and phosphatases that regulate cellular ATG4B activity using siRNA mediated knockdown and cDNA overexpression. Of these, we provide preliminary evidence that the kinase AKT2 enhances ATG4B activity in cells. We provide all raw and processed data from the screens as a resource for further analysis. Overall, our findings provide novel insights into the regulation of ATG4B and highlight the importance of post-translational modifications of ATG4B.

## Introduction

Autophagy is a cellular process central to multiple aspects of health and disease. A key function of autophagy is to mediate lysosomal degradation of cellular material through the formation of an autophagosome, a double-membrane structure that engulfs cytoplasmic material, seals it from the surrounding cytoplasm and delivers it to the lysosome. The formation of an autophagosome is governed by a number of ATG (AuTophaGy-related) proteins that are conserved from yeast to mammalian cells (Tsukada and Ohsumi, 1993).

A key step in the formation of an autophagosome is the conjugation of microtubule-associated protein 1 light chain 3 (LC3) and gamma-aminobutyric acid receptor-associated protein (GABARAP) proteins to the autophagosomal membrane. LC3/GABARAP proteins are synthesized in the cell as an inactive form (pro-LC3/GABARAP) that require activation through C-terminal proteolytic cleavage by the ATG4 family of proteins to generate LC3/GABARAP-I.

It is thought that ATG4 mediates two key processing steps of LC3/GABARAP, the proteolytic processing prior to lipidation and insertion of lipidated LC3/GABARAP-II in the autophagosomal membrane, and the de-lipidation of LC3/GABARAP-II, leading to recycling of processed LC3/GABARAP-I. There are four members of the ATG4 family in mammalian cells that are partially redundant in substrate processing, but have also distinct specificities. ATG4B, the main isoform of the ATG4 family of proteins is regulated by different types of post-translational modifications, including ubiquitination (Kuang et al., 2012), O-GlcNAcylation (Jo et al., 2016), *S*-nitrosylation (Li et al., 2017), capase mediated proteolysis (Betin and Lane, 2009; Betin et al., 2012), redox mechanisms (Scherz-Shouval et al., 2007; Qiao et al., 2015; Heintze et al., 2016) and phosphorylation (Yang et al., 2015; Huang et al., 2017; Pengo et al., 2017; Sanchez-Wandelmer et al., 2017; Ni et al., 2018). It is not well understood how ATG4B hydrolase activity toward its two substrates pro-LC3 and LC3-II could be differentially regulated, but recently it has been pointed out that post-translational modifications may control the ATG4B proteolytic and de-lipidation activity. It has been shown that local phosphorylation by ATG1/ULK1 at the forming autophagosome inhibits ATG4 activity in yeast (Sanchez-Wandelmer et al., 2017) and ATG4B in mammalian cells (Pengo et al., 2017), whereas de-phosphorylation by PP2A renders ATG4B active in the cytoplasm of cells. Other phosphorylation events may also contribute to such regulation, since AKT1 and MST4 are capable of phosphorylating ATG4B (Huang et al., 2017; Ni et al., 2018), although the spatio-temporal context of this has not yet been defined.

A role for ATG4B in cancer has been proposed, including chronic myeloid leukemia (Rothe et al., 2014), osteosarcoma (Akin et al., 2014), colorectal cancer (Liu et al., 2014), prostate cancer (Mouratidis et al., 2014), breast cancer (Bortnik et al., 2016) and pancreatic adenocarcinoma (Yang et al., 2018). The rationale that ATG4 proteins might be therapeutic targets mostly stems from the fact that these proteins are highly over-expressed in some cancer types compared to non-cancerous cells (Costa et al., 2016) and genetic inhibition of ATG4B either through siRNA or use of a dominant negative form of the gene show some benefit in chronic myeloid leukemia (Rothe et al., 2014), breast cancer (Bortnik et al., 2016) and pancreatic carcinoma (Yang et al., 2018).

Multiple efforts are underway to develop biochemical assays to monitor ATG4B activity and thus identify compounds targeting ATG4B (Kurdi et al., 2017). Assay types include the use of enzymatic reporter genes, such as the phospholipase A2-linked substrate approach (Ni et al., 2015), amino-methylcoumarin (AMC)-type esters of LC3 substrates, BRET-based assays (Woo et al., 2014), and gel electrophoresis assays (Cleenewerck et al., 2016).

We have previously developed a cell-based system to monitor cellular ATG4B activity that utilizes the non-conventional secretion of a small luciferase (Ketteler et al., 2008; Luft et al., 2014). Key advantages of this assay are that it is very sensitive, non-invasive and highly quantitative (Ketteler and Seed, 2008). This assay has supported significant discoveries that helped to understand the post-translational regulation of ATG4B. These include the identification of the ubiquitin ligase RNF5 as a key regulator of ATG4B stability (Kuang et al., 2012), the O-GlcNAc modification of ATG4B to increase its proteolytic activity (Jo et al., 2016), and the regulation of ATG4B activity by phosphorylation (Yang et al., 2015; Pengo et al., 2017). Here, we present a small molecule and siRNA screen to identify regulators of ATG4B activity in cell-based assays. The identified compounds are effective to overcome cancer-associated defects in LC3A processing and are valuable tool compounds for further development and understanding of ATG4 biology. Furthermore, we have identified a number of kinases that modulate ATG4B-mediated LC3 processing that were not previously known to have this function.

## Materials and Methods

### Cell Lines

HeLa cells expressing mCherryLC3 were obtained from Dr. Ramnik Xavier (Massachusetts General Hospital, Boston). Retroviral supernatants of HEK293T cells transfected with pMOWS-ActinLC3dNGLUC, GagPol and VSV-G were obtained by calcium phosphate precipitation as described (Ketteler et al., 2002) to generate stable HeLa ActinLC3dNGLUC/mCherryLC3 and HEK293T-ActinLC3dNGLUC cells. Briefly, pBABE-mCherry-GFP-LC3 or pMOWS-ActinLC3dN was transfected with VSV-G and GagPol into HEK293 cells using calcium phosphate transfection. Supernatants were harvested and filtered through 0.45 μm filters, supplemented with 8 μg/ml final concentration of polybrene (Sigma) and added to target cells for overnight incubation. Transduced cells were then passaged and selected with puromycin. All cell lines were cultured in Dulbecco’s Modified Eagle Medium (DMEM) supplemented with 10% fetal calf serum (FCS, Life Technologies), L-Glutamine and Penicillin/Streptomycin.

### Plasmids

Expression plasmids pGEXATG4B and pGEXATG4B^C74S^ were created as described previously (Pengo et al., 2017). pGEXATG4B^Δ1-24^ (mutation by deletion of 24 amino acid residues from the N-terminus), pGEXATG4B^S34A^, pGEXATG4B^S34D^ and pGEXATG4B^S121A^ were created by PCR using pGEXATG4B as a template and primers Δ1–24 forward (5′-CCC GTT TGG ATA CTG GGT AGA AAA TAC AGC-3′) and Δ1–24 reverse (5′-GAA TTC CGG GGA TCC CAG GGG C-3′), primers S34A forward (5′-GCT ATT TTC ACA GAA AAG GAC GAG-3′) and S34A reverse (5′-GTA TTT TCT ACC CAG TAT CCA AAC-3′), primers S34D forward (5′-GAT ATT TTC ACA GAA AAG GAC GAG-3′) and S34D reverse (5′-GTA TTT TCT ACC CAG TAT CCA AAC-3′), primers S121A forward (5′-GCT TAC TAC TCC ATT CAC CAG ATA-3′) and S121A reverse (5′-GTC CTT CCT GTC GAT GAA TGC GTT-3′), and primers S262A forward (5′- GCA GCC CAC TAC TTC ATC GGC TA-3′) and S262A_reverse (5′- GTT GGG CTT CCC TCC GAT GAC-3′), respectively. All PCR were performed at 30 cycles using Pyrobest DNA polymerase (Takara, R005A). The PCR products were phosphorylated, ligated and transformed into *Escherichia coli* DH5α for selection of correct plasmids. The following constructs were described elsewhere: pMOWS-ActinLC3dN (Ketteler et al., 2008), pGEXCLK2cd (Prak et al., 2016), pEAK12-ActinLC3A-R70H-dNGLUC (Costa et al., 2016), and pEAK12-GFP (Ketteler et al., 2008). The ATG4B promoter construct ATG4B-FLUC was obtained from Switchgear Genomics (#S711306). The sequence of the inserted promoter region from ATG4B is shown in Supplementary Figure S1. The vector map can be found on the company’s website ^1^. Transcriptional activation of ATG4B promoter was measured by monitoring Renilla luciferase in cell lysates according to the manufacturer’s instructions (Promega).

### Compounds

The Chemibank compound collection was obtained from David Selwood ^2^. The ranges of some of the molecular properties are as follows: molecular weight between 126 and 600, AlogP between -3.5 to 6, hydrogen bond donors between 0 and 6, hydrogen bond acceptors between 0 and 12, rotable bonds between 0 and 15 and number of rings between 1 and 8. The library has hit-like properties (rule of 6) and falls just outside the Lipinski’s rule of five. A total of 30,000 compounds was stored as a 10 mM DMSO stock solution under nitrogen (5% O_2_) and low humidity (5%) at room temperature and in the dark (Roylan San Francisco storage pod). For screening, compounds were transferred to assay plates using the Labcyte Echo 520 at a final concentration of 10 μM with a final DMSO concentration of 0.2%. Hit compounds were re-purchased from Asinex (Delft, Netherlands), or Life Chemicals (Ukraine). Bafilomycin A, DTT, H_2_O_2_, *N*-acetyl cysteine and rapamycin were obtained from Sigma.

### Small Molecule Screening

High-Throughput Screening was performed in 384-well plates (Greiner). First, compound was added to the plates using the Echo 520 (Labcyte). Next, HeLa-ActinLC3dNGLUC cells (20,000/well) were dispensed onto the compounds using the Thermo Fisher Multidrop and cultured for 24 h at 37°C. Supernatants (5 μl) were harvested and dispensed into black 384-well plates. Native coelenterazine (Cambridge Bioscience, #BT10110) in GLUC buffer (0.1% disodium phosphate, 5% glycerol, 150 mM sodium bromide, 1 mM EDTA, 25 mM Tris-HCL pH8 and 2 mM ascorbic acid) at a final concentration of 10 μg/ml was injected immediately prior to analysis using the Envision II (PerkinElmer) plate reader. For *Z*′ factor calculation, the following formula was used:

$$Z\prime =1-(3\text{x}({\text{STD}}_{\text{Pos}}+{\text{STD}}_{\text{neg}})/|{\text{mean}}_{\text{pos}}-{\text{mean}}_{\text{neg}}|)$$

with STD_pos_ = standard deviation of the positive control and STD_neg_ = standard deviation of the negative control. For cell-based assays, we accept values that are higher than 0.3 for the *Z*′ factor.

### siRNA and cDNA Screening

Stable HEK293T-ActinLC3dNGLUC were sent for STR profiling and confirmed as HEK293T cells. Cells were counted and 5,000 cells were seeded into 384-well and incubated overnight at 37°C and 5% CO_2_. The siRNA library for human kinases and human phosphatases (Sigma MISSION, Supplementary File S1) consists of 3 siRNA oligonucleotides per gene in a 96-well format where the outer columns 1 and 12 were used for controls. First, the 3 siRNAs for each gene were pooled using the automated Tecan Freedom Evo liquid handler. The siRNA pools were then transfected at a final concentration of 55 nM with lipofectamine 2000 (Invitrogen) using an automated protocol on the Tecan Freedom Evo in 384-well plates. Briefly, 5 μl of the siRNA stock solution (100 μM) was mixed with 0.5 μl lipofectamine and 50 μl Optimem for 20 min at RT. Ten μl of this mixture was added to cells in the 384-well plate to a total volume of 50 μl and incubated for 48 h at 37°C. After 48 h, 5 μl of supernatant was transferred using the Tecan Freedom Evo to black 384 multi-well plates and 25 μl substrate of native coelenterazine was added prior to reading luminescence in the PerkinElmer Envision II. Substrate was added using the injectors of the PerkinElmer Envision II to ensure equal times from addition of substrate to measurement in all wells. The cDNA kinome library (Supplementary File S2; Thermo Fisher) was transfected at 100 ng/well in HEK293T cells stably expressing the ActinLC3dNGLUC reporter and luciferase release was monitored after 24 h.

### Statistical Analysis

The primary screening data was analyzed using CellHTS2 (Boutros et al., 2006). Relative luciferase light units were normalized across the plate and the *B* scores were calculated to determine Z scores of each individual compound. All error bars shown unless otherwise indicated are calculated as standard deviations from the mean of the replicates. Statistical significance was calculated using a two-sided paired *T*-Test (Microsoft Excel). In Figure 3C, a one-way ANOVA with Tukey’s multiple comparison test was applied to calculate significances. The graph was drawn in GraphPad Prism.

### Luciferase Release Assay

The luciferase release assay was described previously (Ketteler et al., 2008). Native coelenterazine was prepared as 1 mg/ml stock solution in acidified Methanol and diluted 1:100 in PBS or GLUC assay buffer. Typically, five μl of supernatant was harvested and mixed with 25 μl coelenterazine in 384-well plates or 50 μl of coelenterazine in 96-well plates. All experiments were performed in triplicates except the siRNA screen that was done in quadruplicates.

### Cell Viability Assay

Cellular Viability was assessed using the Cell Counting Kit (CCK8, Sigma). Briefly, 5 μl of CCK8 solution was added in 50 μl PBS to the cells and incubated for 60 min at 37°C prior to measurement of absorbance at 450 nm in the Envision II.

### Protein Purification

Recombinant proteins were purified from bacteria as described previously (Prak et al., 2016). Protein expression and purification of LC3B-GST, ATG4B, and ATG4B mutant C74S was done as described previously (Pengo et al., 2017). Protein expression and purification of ATG4B mutants Δ1–24, S34A and S34D were done the same way as that of ATG4B. GST was removed from GST-tagged ATG4B and GST-tagged ATG4B mutants using PreScission Protease (GE Healthcare, 27-0843-01). All recombinant proteins were stored at -80°C in 50 mM Tris-HCl, pH 8.0, 150 mM NaCl, 0.5 mM EDTA, 0.1 mM EGTA, 33% glycerol and 1 mM dithiothreitol (DTT).

### *In vitro* Phosphorylation Assays

*In vitro* radioactive assays were performed by incubating 100 ng recombinant ATG4B diluted in assay buffer (20 mM Tris-HCl pH 7.5, 10 mM MgCl_2_, 5 mM DTT, 20 μM cold ATP, 0.16 μM ATP [γ-^32^P] Perkin-Elmer NEG502A100UC) in the presence of recombinant AKT2 (Sigma-Millipore) at 30°C for the indicated time. The reaction was stopped by adding 5X SDS Loading Buffer and boiling for 5 min. Samples were loaded on NUPAGE Acrylamide gel (Invitrogen, NP0321BOX). Gels were stained with InstantBlue Protein Stain (Expedeon, ISB1L) before drying on filter paper and measuring incorporated radioactivity by exposing on photographic film (Bio-Rad).

### LC3B-GST Cleavage Assay and Analysis of Enzyme Kinetics for ATG4B and Its Mutants

The cleavage assay was done at 37°C in a reaction volume of 20 μl containing 1 mg/ml LC3B-GST and 0.004 mg/ml ATG4B wide type and mutants Δ1–24, S34A and S34D in assay buffer A (50 mM Tris-HCl pH 8.0, 150 mM NaCl, 5 mM DTT) for 0.4–10 min. The reaction was stopped by adding the same volume of 2X SDS Loading Buffer and boiling for 5 min. The sample were analyzed on a 4–20% Mini-PROTEAN^®^ TGX^TM^ Precast Gel (Bio-Rad, 456 1096) and Coomassie Brilliant Blue Staining. The images of the gels were scanned and the intensity of each protein band were quantified using Fiji^3^ using the analyse >gel built-in function. The percentage of the substrate that remain at each reaction time point (% of remaining substrate, *y*-axis) equal to optical density (OD) LC3-GST/(OD LC3-GST + OD GST + OD LC3-I) × 100% was plotted versus the reaction time (s, *x*-axis) and the curves were then fitted using the non-linear regression method in R software, from which the time needed to catalyze amount of substrate were derived.

To analyze the enzyme kinetics for ATG4B and its mutants, purified ATG4B and ATG4B mutants at 45.15 nM except mutant C74S at 11.3 μM were incubated with twofold serial dilutions of LC3B-GST from 39 to 2.4 μM in assay buffer A in a reaction volume of 20 μl at 37°C. The incubation time was 6 min for all except mutant C74S was incubated for 5 h. The reaction was stopped by adding the same volume of 2X SDS Loading Buffer and boiling for 5 min. The samples were subjected to a 4–20% Mini-PROTEAN^®^ gel and the intensity of protein bands were analyzed the same way as that of the cleavage assay above. The initial velocity (μM/min, *y*-axis) was calculated as the concentration of GST produced, which was plotted versus the concentration of substrate LC3B-GST before reaction (μM, *x*-axis). The curves were then fitted using the non-linear regression method in R software, from which the *V*_max_ and *K*_m_ (Michaelis constant) for each enzyme-substrate reaction were derived. The *k*_cat_ (catalytic constant) was determined diving *V*_max_ by the enzyme concentration. The catalytic efficiency is defined as *k*_cat_/*K*_m_ (inverse molar liter per second).

## Results

### Optimization of a Cell-Based ATG4B Sensor for High-Throughput Screening

**FIGURE 1:**
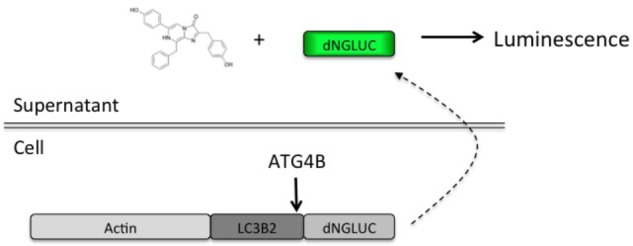
Schematic of the luciferase release assay. A fragment of Gaussia luciferase is linked to full-length LC3B2 and β-actin inside cells. Upon cleavage of LC3B2 by ATG4B, Gaussia luciferase (dNGLUC) is released into the supernatants and can be monitored as a correlate of cellular ATG4B activity. The structure of native coelenterazine is shown.

In order to set up a screen for small molecule regulators of ATG4B, we used the previously described luciferase release assay (Ketteler et al., 2008). This assay relies on non-conventional release of Gaussia luciferase (GLUC) from cells upon ATG4B-dependent cleavage of an ActinLC3B2dNGLUC reporter construct (Figure 1). The amount of luciferase in supernatants correlates with cellular ATG4B activity, making this a very simple quantitative assay. We have recently confirmed that non-conventional release of GLUC from cells is not dependent on autophagosome formation, since ATG5 knockout cells are able to release GLUC from cells (Luft et al., 2014). Thus, this assay is suitable for screening for modulators of ATG4B-mediated LC3 cleavage.

**FIGURE 2:**
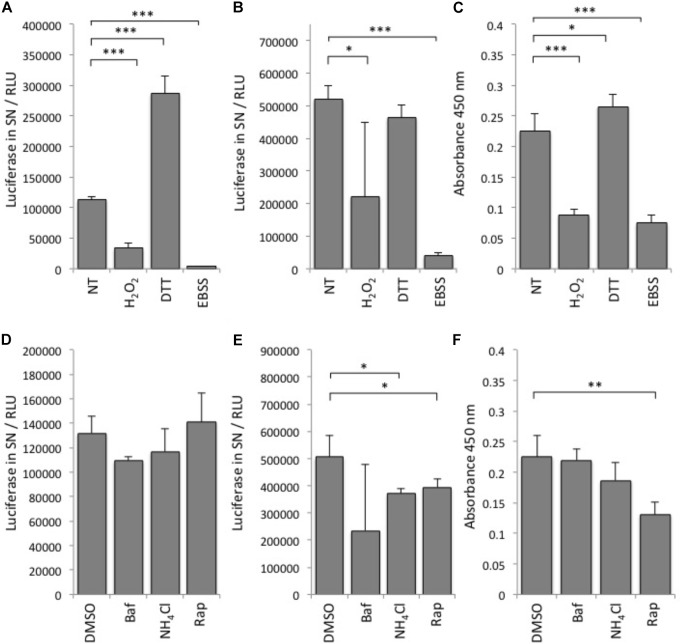
Performance of the luciferase release assay. **(A)** Luciferase activity in relative light units (RLU) was monitored in supernatants (SN) of HEK293T cells transfected with ActinLC3dNGLUC and treated with the indicated reagents or left (NT, not treated). **(B)** HEK293T cells transfected with dNGLUC (without ActinLC3 linker) were treated as in **(A)** and luminescence released into supernatants was measured as an indicator of general dNGLUC secretion. **(C)** Cell viability was measured using the cell-counting kit 8 (CCK-8, Sigma). The same cells as in **(A)**, were subjected to the CCK-8 cell viability assay by adding the cell viability reagent for 60 min before reading the absorbance at 450 nm in the PerkinElmer Envision II. Lower absorbance values reflect low cell viability. **(D)** HEK293T cells were transfected with ActinLC3dNGLUC and treated with the indicated reagents or DMSO as control. **(E)** HEK293T cells transfected with dNGLUC (without ActinLC3 linker) were treated as in **(D)** and luminescence released into supernatants was measured in the PerkinElmer Envision II. **(F)** Cell viability was measured as in **(C)** after treatment with the indicated reagents. Baf, bafilomycin A; Rap, rapamycin. All results displayed are from three independent experiments and statistical significance was determined using a two-sided paired *T*-Test (^∗∗∗^*p* < 0.001, ^∗∗^*p* < 0.01, and ^∗^*p* < 0.05). Error bars represent standard deviation.

First, we tested various autophagy-modulating compounds for response in the luciferase release assay (Figure 2). In line with previous observations that ATG4B activity is highly redox-dependent (Scherz-Shouval et al., 2007), we found that treatment of cells with DTT can strongly activate the reporter, whereas treatment with H_2_O_2_ reduced reporter activity (Figure 2A). To assess whether the treatment affected general dNGLUC secretion, we expressed dNGLUC, which is constitutively released independent of ATG4B. We observed that DTT did not modulate secretion of dNGLUC and H_2_O_2_ moderately reduced secretion (Figure 2B). This decrease in dNGLUC secretion was due to strongly reduced cell viability upon treatment with H_2_O_2_ whereas treatment with DTT only mildly affected cell viability (Figure 2C). Overall, these results suggest that redox regulation directly affects cellular ATG4B activity. Other autophagy modulating treatments such as NH_4_Cl or bafilomycin A1 had very little impact on cellular ATG4B activity (Figure 2D), confirming that the luciferase release assay specifically measures ATG4B activity and not general autophagy activity or flux. Starvation by EBSS resulted in a decrease in luciferase secretion from the ActinLC3dNGLUC construct, but at the same time a decrease in general dNGLUC secretion and cell viability (Figures 2A–C) as well, suggesting that the decrease in luciferase release is due to reduced cell viability. Treatment with rapamycin had no strong effect on luciferase release from the ActinLC3dNGLUC reporter, but reduced both overall dNGLUC secretion and cell viability (Figures 2D–F). Upon calculating the ratio of luciferase release over cell viability, rapamycin was confirmed as inducer of ATG4B activity (Table 1). In conclusion, the ATG4B luciferase release assay specifically detects cellular ATG4B activity such as redox-sensitive mechanisms. One caveat in using this assay is that effects on cellular viability can reduce the net release of luciferase from the reporter, but such effects can be normalized by assessing the cellular viability in parallel.

**Table 1:** Ratios of luciferase released from cells expressing the ActinLC3BdNGLUC reporter normalized by cell viability.

Treatment	Ratio Luc/viability
DMSO	1.00 ± 0.26
DTT	1.84 ± 0.32
H_2_0_2_	0.66 ± 0.24
Baf	0.85 ± 0.10
EBSS	0.09 ± 0.04
NH_4_CI	1.07 ± 0.35
Rapamycin	1.84 ± 0.59

*Data calculated from values in Figure 2. Displayed are mean values with standard deviation*.

Next, we established that the assay is amenable to high-throughput screening by determining the *Z*′ factor, a good surrogate for assessing the robustness of an assay (Zhang et al., 1999). In the absence of potent small molecule inhibitors or activators of ATG4B, we used Brefeldin A as a well characterized inhibitor of dNGLUC secretion (Luft et al., 2014). As shown in Supplementary Figure S2, Brefeldin A resulted in a robust reduction in secreted dNGLUC from cells. We determined the *Z*′ factor as 0.46, which was within a suitable range for cellular screening. We screened a collection of 30,000 compounds from UCL Chemibank ^4^ in 384-well format in triplicates. The raw luminescence values in the supernatants were normalized to the plate median and a B score analysis was applied to account for possible edge effects (Supplementary Figure S2).

**FIGURE 3:**
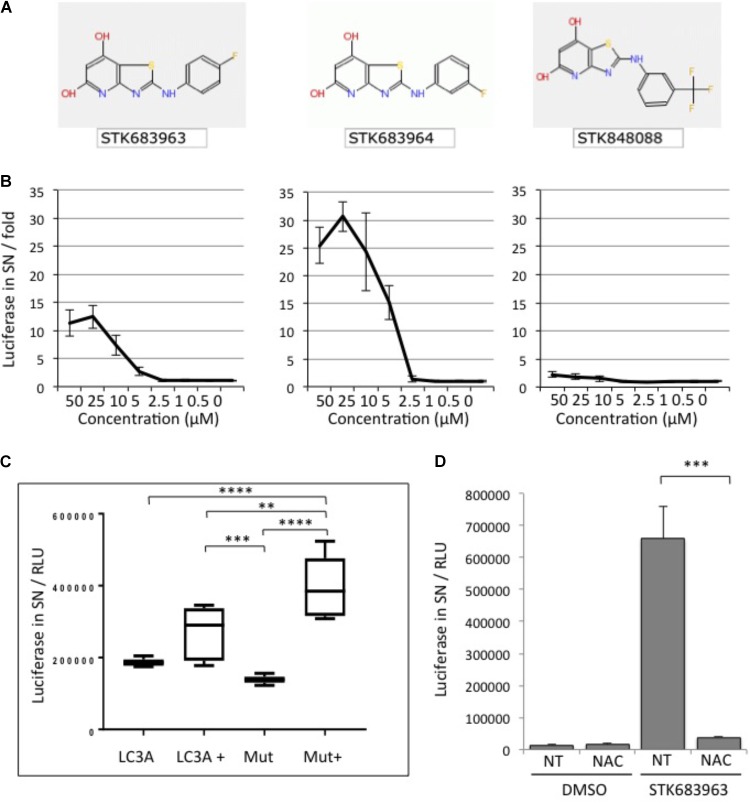
STK683963 is a novel activator of ATG4B. **(A)** Structures of hit compound STK683963 and its analogs STK683964 and STK848088. **(B)** HeLa-ActinLC3dNGLUC cells were treated with the hit compound STK683963 and two analogs (STK683964, STK848088) overnight at the indicated concentrations and luciferase activity released into supernatants was monitored. Data expressed as fold of DMSO control. **(C)** HEK293T cells were transfected with ActinLC3AdNGLUC or ActinLC3A-R70H-dNGLUC and treated overnight with DMSO or STK683963. Luciferase release was measured in the PerkinElmer Envision II. A one-way ANOVA with Tukey’s multiple comparison test was applied to calculate significances (^∗∗∗∗^*p* < 0.0001 and ^∗∗^*p* < 0.01). **(D)** HeLa cells stably expressing ActinLC3dNGLUC were treated with STK683963 in combination with *N*-acetyl cysteine (NAC) and luciferase release was monitored after an overnight incubation. NT, not treated. All results displayed are from three independent replicates and statistical significance was determined using a two-tailed paired *T*-Test (^∗∗∗^*p* < 0.001 and ^∗^*p* < 0.05). Error bars represent standard deviation.

A strong activator of cellular ATG4B activity identified in the screening was the compound STK683963 (Figure 3A). STK683963 strongly up-regulates the luciferase release reporter in a dose-dependent manner (Figure 3B) after 24 h. The analogous compound STK683964 showed slightly higher luciferase values in a similar concentration range in activating ATG4B, whereas another analog STK848088 did not. The effect of STK693963 on increasing ATG4B activity is most likely indirect since the increase in luciferase was only seen after overnight treatment and not at earlier time points. Also, STK693963 had no effect on *in vitro* ATG4B-mediated cleavage of a LC3B-GST reporter (data not shown). One possibility was that the compound might enhance ATG4B transcription. However, STK683963 does not activate ATG4B transcript expression since we could not observe an increase in the transcriptional activation of an ATG4B-promoter-luciferase construct (Supplementary Figure S3), while the positive control, Biochanin A, resulted in an increase in luciferase expression. STK683963 had no effect on viability of HeLa cells (Supplementary Figure S4). Next, we tested whether STK683963 can overcome LC3A deficiency that is associated with the R70H cancer mutation (Costa et al., 2016). When LC3A R70H was inserted in the ActinLC3dNGLUC reporter to monitor cleavage of this mutant, we observed that ATG4B-mediated processing was mildly reduced (Figure 3C). However, treatment with STK683963 activated LC3A R70H processing, suggesting that it can be used to enhance LC3 processing deficiencies in some conditions. In order to identify a possible mechanism of action for STK6983963, we investigated whether it might act on the redox mechanism of ATG4B. Thus, we treated cells with *N*-acetyl cysteine (NAC), a reducing agent that was previously shown to affect LC3 processing (Heintze et al., 2016). We found that STK683963 strongly activated luciferase release, which was completely blocked in the presence of NAC (Figure 3D). Thus, we propose that STK683963 acts as a mediator of redox-regulation of ATG4B in cells and is a strong activator of cellular ATG4B activity.

### siRNA Screening to Identify Regulators of ATG4B Activity

**FIGURE 4:**
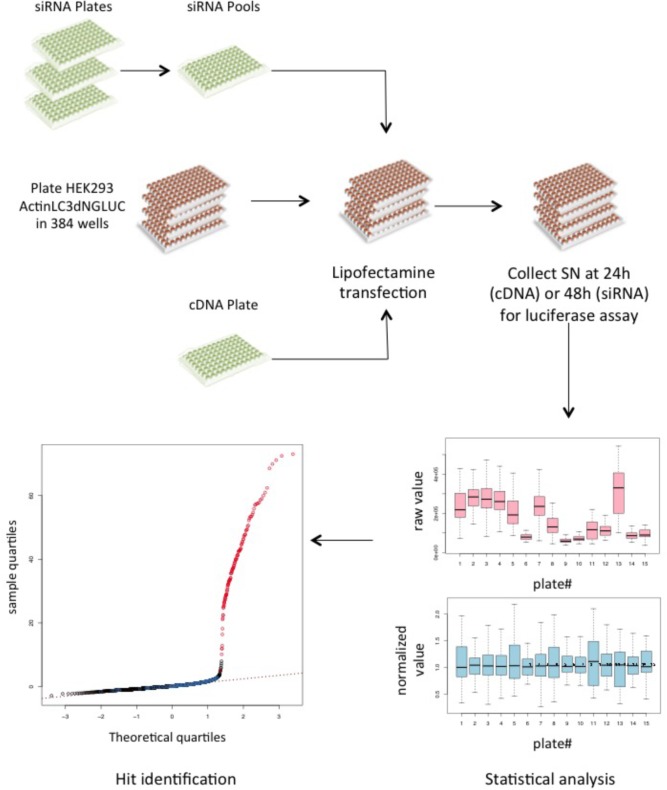
A cDNA and siRNA screen to identify kinases and phosphatases that regulate ATG4B activity. Schematic of the screening workflow. For each gene, three different siRNA oligonucleotides were pooled together in each well and a transfection mix was dispensed into 384-well plates, where each quadrant corresponds to one 96-well layout. For cDNA transfection, the library was re-arrayed from 96-well plates into 384-well plates and transfected onto the reporter cell line. A total of 15 siRNA plates (11 kinase and 4 phosphatase plates) were screened in quadruplicates and the cDNA library was screened in triplicates. Supernatants (SN) were collected 48 h after transfection and measured on the PerkinElmer Envision II. All raw values were put into CellHTS2 for statistical analysis and hits were identified based on ranking of the normalized values.

Having established that the luciferase reporter is amenable to large-scale screening, we reasoned that this assay is well suited to identify regulators of ATG4B activity in cells. We therefore screened siRNA libraries targeting the human kinome and human phosphatome and a cDNA overexpression library of human kinases in HEK293T cells stably expressing the luciferase release construct (Figure 4). By reverse transfection, we seeded HEK293T-ActinLC3dNGLUC cells into 384-well plates on top of the siRNA transfection mix using an automated workflow. As a negative control, cells were left untransfected, and as a positive control, we transfected cDNA expressing ATG4B in the last column. The cells were incubated for 48h to achieve knockdown of the target genes, prior to harvesting the supernatants and analysis of luciferase activity in the PerkinElmer Envision II plate reader. Raw values were normalized to plate median and ranked by *Z* score. The robustness of the screen was assessed in four replicates, and overall standard deviations showed that the results were highly reproducible. The code used in R package for analysis is shown in Supplementary File S3. The complete set of results from the siRNA screen displaying activators and inhibitors is shown in Supplementary File S4. Several strong inhibitors of ATG4B activity were observed (knockdown of these genes resulted in an increase of luciferase activity). These include VRK1, TYK2, TRIB1, STK11, GUCY2B, and CAMK2D with a significant *Z* score above 4 (Table 2). Interestingly, CAMK2D was previously reported in another siRNA screen as inhibitor of LC3 puncta formation (Szyniarowski et al., 2011), in agreement with our results and suggesting it may control pro-LC3 processing. The strongest inhibition was seen upon knockdown of PAK1 (*Z* score = -2.85). Overall, a higher number of genes resulting in activation upon knockdown than inhibition were identified.

**Table 2:** Hits from siRNA screening.

Gene name	*Z* Score
VRK1	7.97
TYK2	7.22
TRIBl	6.66
STK11	5.89
GUCY2D	5.87
CAMK2D	5.18
SNRK	5.07
PPP1R9B	4.89
AKAP13	4.78
MAST3	4.03

*The top 10 inhibitors, where knockdown increases luciferase release from cells are shown and their Z score after normalization calculated from the median of four replicates. For full details including statistics, see Supplementary File S4*.

### AKT2 Activates ATG4B-Mediated LC3 Processing

**FIGURE 5:**
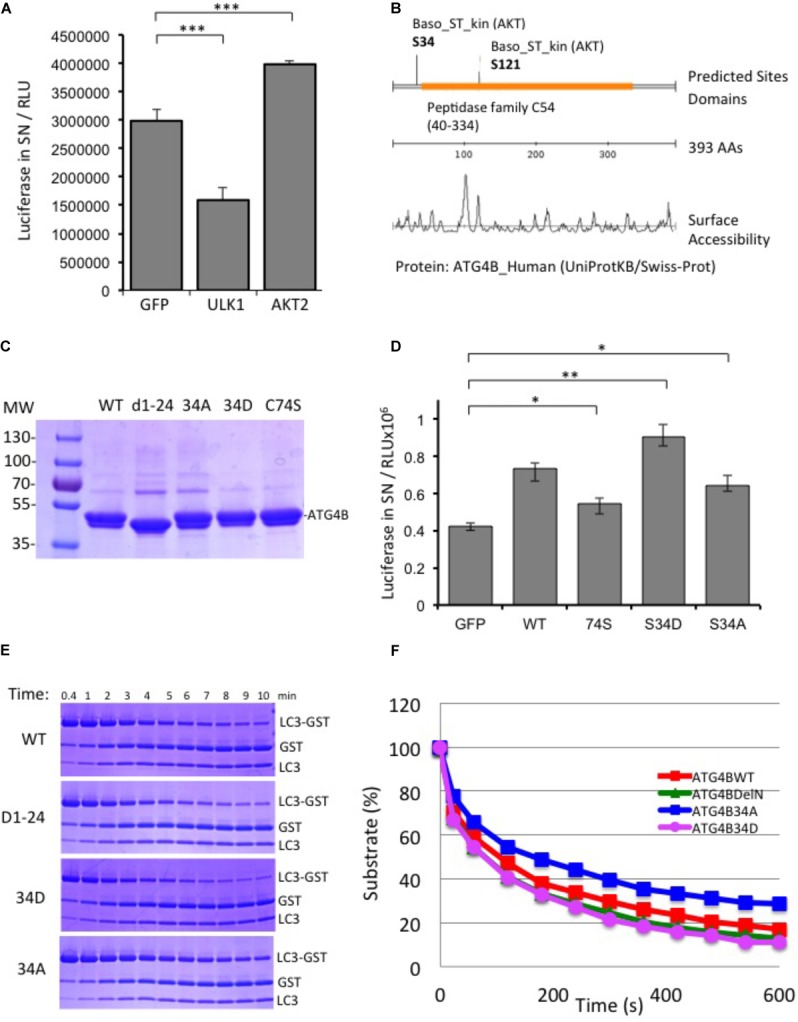
AKT2 activates ATG4B. **(A)** HEK293T cells stably expressing ActinLC3dNGLUC were transfected with cDNA for ULK1 or AKT2 and luciferase activity was monitored as described. ULK1 strongly inhibited luciferase release, while AKT2 activated the reporter. Results displayed are from three independent replicates and statistical significance was determined using a two-sided paired *T*-Test (^∗∗∗^*p* < 0.001). **(B)** Prediction of AKT2 phospho-target sites from Scansite (www.scansite.mit.edu). The ATG4B protein sequence (UniProt ID Q9Y4P1) was used as input and prediction was performed at low stringency. Serine 34 and Serine 121 were predicted as potential phosphorylation sites for AKT2. **(C)** Coomassie gel of the purified proteins. Equal amounts of the proteins were used as input in the following experiments. **(D)** HEK293T cells stably expressing ActinLC3dNGLUC were transfected with pEAK12-GFP as a control, or wild-type (wt) ATG4B and its mutants (C74S, S34D, and S34A). Luciferase activity in supernatants was monitored as described (^∗∗^*p* < 0.01 and ^∗^*p* < 0.05). **(E)** GST-LC3 cleavage assay to determine time kinetics for 0.004 mg/ml of ATG4B and mutants (without GST-tag) to catalyze 50% of 1 mg/ml substrate LC3B-GST (see section “Materials and Methods”). **(F)** The catalytic kinetics were determined after incubation of the purified enzyme with LC3B-GST at 37°C from three different experiments and % of remaining substrate is shown.

In parallel to the siRNA screen, we also performed a cDNA overexpression screen using the luciferase reporter assay (Supplementary Figure S5). For cDNA expression screening, we combined the four 96-well plates of the human kinome library into one 384-well plate and transfected this in triplicates in HEK293T-ActinLCdNGLUC cells. We identified a number of activators and inhibitors of ATG4B-mediated luciferase release (Supplementary File S5). We were particularly interested in AKT2 since AKT1 has previously been shown to be involved in autophagy and mitophagy (Ni et al., 2018; Soutar et al., 2018). First, we transfected AKT2 in the ActinLC3dNGLUC reporter cell line, concomitantly with ULK1, a kinase we recently identified as a negative regulator of ATG4B activity and measured luciferase release (Pengo et al., 2017). As expected, ULK1 significantly reduced ATG4B-mediated LC3 processing, whereas AKT2 overexpression activated the luciferase reporter (Figure 5A). In order to determine potential phosphorylation sites in ATG4B, we used the scansite algorithm to search for AKT2 target sites (Obenauer et al., 2003). Scansite can generate predictions of protein residues that are phosphorylated by protein kinases based on data derived from experimental peptide arrays. Two sites in ATG4B were predicted as potential AKT2 target site, Serine 34 and Serine 121 (Figure 5B). One of these sites, Serine 34 was previously reported as a target of AKT1-mediated phosphorylation (Ni et al., 2018). Therefore, we generated mutants of ATG4B that either cannot be phosphorylated (S34A) or that mimic constitutive phosphorylation (S34D) (Figure 5C) and investigated the consequence of S34A and S34D mutation on cellular ATG4B activity. Indeed, ATG4B S34A showed reduced ATG4B activity in the luciferase release assay, although higher than the catalytic mutant C74S, whereas S34D showed higher activity than WT ATG4B (Figure 5D), in line with a potential role for AKT in positively regulating the activity of ATG4B.

Additionally, we saw that both an N-terminal deletion mutant (Δ1–24) and the S34D mutant exhibited increased LC3 substrate cleavage *in vitro* using the LC3-GST assay (Figure 5E). The calculation of the *K*_m_ values of mutants compared to ATG4B WT was 2.15, 1.74, 1.86, and 2.55 × 10^-5^ for ATG4B WT, Δ1–24, S34D and S34A, respectively (Figure 5F and Table 3). Overall, these results suggest that post-translational modification of Serine 34 in ATG4B might influence the LC3B processing kinetics.

**Table 3:** Enzyme kinetics of *in vitro* cleavage assay.

	*K*_m_ (M)	STDV	*K*_cat_/*K*_m_ (1/M.S)	STDV
WT	2.14957E-05	3.21287E-07	73758.29226	1955.269708
Δ1-14	1.74178E-05	4.47812E-06	86959.80316	1099.428911
34A	1.86258E- 05	4.51334E-06	65214.59464	7583.879041
34D	2.54884E-05	6.56644E-06	83324.94999	5185.885677

*The indicated ATG4B mutants were incubated with GST LC3B substrate and subjected to in vitro cleavage as described in the “Materials and Methods” section*.

**FIGURE 6:**
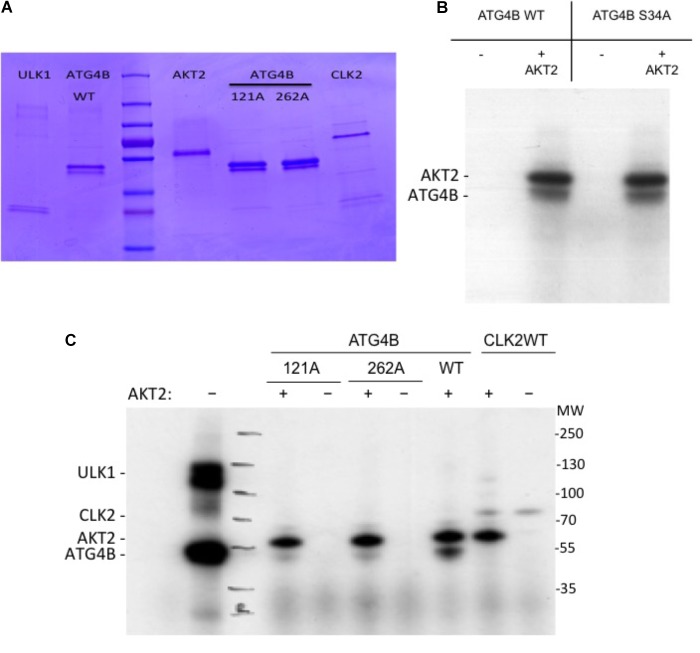
*In vitro* kinase assay. **(A)** Coomassie gel showing the purified proteins used in the *in vitro* kinase activity assays. **(B)** Recombinant ATG4B or ATG4B S34A was incubated with recombinant AKT2 and ATP γ-^32^P and incorporation of labeled γ-^32^P was measured by auto-radiography. The upper band corresponds to AKT2 auto-phosphorylation and the lower band corresponds to ATG4B. **(C)** Recombinant ATG4B wild-type (WT), S121A or S262A were incubated with (+) or without (–) recombinant AKT2 and incorporation of labeled γ-^32^P was measured by auto-radiography. On the left side, ULK1 mediated phosphorylation of ATG4B was included as control. On the right side, CLK2 (CLK2 catalytic domain with GST-tagged (Prak et al., 2016)) was included as another protein control to show that in the absence of ATG4B, AKT2 resulted in auto-phosphorylation.

Next, in order to assess whether ATG4B can be phosphorylated by AKT2, we performed an *in vitro* kinase assay (Figure 6). In addition to ATG4B S34A, we also generated two other mutants, ATG4B S121A and S262A (Figure 6A). We detected a phosphorylation signal in the presence of AKT2 but not in the presence of another control kinase (CLK2) indicating that AKT2 can phosphorylate ATG4B *in vitro* (Figures 6B,C). However, phosphorylation was also evident in the ATG4B S34A mutant, suggesting that there are other phosphorylation sites in ATG4B. Indeed, when assaying the S121A and S262A mutants, we observed a strong decrease in phosphorylated ATG4B S121A and S262A, indicating that these sites might be targets for AKT2, at least *in vitro* (Figure 6C). Overall, our results suggest that multiple sites in ATG4B can be phosphorylated by AKT2, all potentially contributing to the regulation of activity.

## Discussion

The autophagy machinery has the delicate task to co-ordinate the initiation and formation of an autophagosome under basal conditions and upon stresses that induce autophagy. How these events – from initiation to fusion with the lysosome – are controlled is only poorly understood. It has recently been suggested that the spatio-temporal control may be regulated by post-translational modification of specific autophagy proteins (Pengo et al., 2017; Sanchez-Wandelmer et al., 2017). Therefore, we set out to get a better understanding of the post-translational regulation of ATG4B, one of the key enzymes that co-ordinate the processing of LC3/GABARP family of proteins.

Multiple factors that regulate ATG4B activity are starting to emerge. A key regulator of ATG4 activity is redox-regulation, which was initially shown to modulate the de-lipidation reaction (Scherz-Shouval et al., 2007). It is now well established that oxidation of a cysteine residue in proximity to the catalytic site reduces LC3 processing. How this redox regulation is established in cells is less well understood, but it is thought that sub-cellular areas of increased ROS production may specifically affect local ATG4 activity. In line with this, thioredoxin and NADPH regulating enzymes such as Ribose-5-phosphate isomerase have been linked to ATG4-mediated LC3 processing (Perez-Perez et al., 2014, 2016; Heintze et al., 2016). It is thus not surprising that compounds that modulate redox signaling may affect cellular ATG4B activity. However, no small molecule compound activator of ATG4B has been identified to date. Here, we have shown that STK683963 and analogs are strong activators of cellular ATG4B activity. We only observed activation after overnight treatment and not at earlier time points (data not shown), suggesting that the compound may not act directly on ATG4B, but rather through indirect mechanisms. Furthermore, treatment with NAC – a reducing agent – blocked the activation of cellular ATG4B activity, hypothesizing that STK683963 primarily acts through a redox-regulated mechanism. The identification of this ATG4B enhancing compound provides a very useful tool that may have applications in conditions where reduced ATG4 activity or reduced LC3 processing has been observed (Costa et al., 2016).

In addition to redox mechanisms, phosphorylation of ATG4 family members is emerging as an important step in the regulation of cellular autophagy. The first evidence for this concept came from the observation that two residues, Ser383 and Ser392, were phosphorylated in cells, but the underlying kinase responsible has not been identified (Yang et al., 2015). In addition, multiple kinases are known to directly regulate and/or bind to ATG4B: ULK1/ATG1 mediated phosphorylation reduces ATG4B activity in mammalian cells (Pengo et al., 2017) and ATG4 activity in yeast (Sanchez-Wandelmer et al., 2017). AKT1 can bind to and phosphorylate Ser34 in ATG4B (Ni et al., 2018), but the effects on ATG4B activity have not been fully addressed. Recently, MST4 has been shown to phosphorylate Ser383 (Huang et al., 2017). Overall, these findings point to a complex regulation of ATG4B activity by kinases, and it is possible that such phospho-regulation may be dependent on the sub-cellular localisation of the kinase (Sanchez-Wandelmer et al., 2017).

High-throughput screens to identify regulators of autophagy have previously been performed. The screens published to date utilize siRNA libraries in phenotypic assays, studying the formation of the autophagosome either through immunostaining or a fluorescent protein reporter linked to LC3 (Chan et al., 2007; Lipinski et al., 2010a,b; Szyniarowski et al., 2011; McKnight et al., 2012). Here, we present the first siRNA- and cDNA-based screen that interrogates the function of ATG4B, using a luciferase-based readout. We have identified multiple kinases and phosphatases that regulate ATG4B activity. In particular, AKT2 is a novel gene that activates ATG4B, and promises to be an interesting candidate for future studies. The AKT family of proteins are known to regulate autophagosome formation and mitophagy (Soutar et al., 2018), and AKT1 has recently been shown to directly phosphorylate ATG4B at Ser34 (Ni et al., 2018). However, it has not been assessed whether this phosphorylation resulted in an increase or decrease of ATG4B activity. In agreement, we identified AKT2 as an activator of ATG4B in our cDNA expression screen. Of note, the two homologs AKT1 and AKT3 were not present in the cDNA library that we used. We noted that a S34A mutant displayed reduced ATG4B activity, while a S34D phospho-mimetic mutant showed an increase in ATG4B activity (Figure 5). We identified other potential AKT2-mediated phosphorylation sites within ATG4B at serine 121 and serine 262. Our assays do not rule out the phosphorylation of serine 34, since this may be masked by the two other sites in our assay. However, at this point we cannot attribute the AKT2-mediated activation of cellular ATG4B activity to a single phosphorylation site within ATG4B. Overall, our findings point to a complex level of regulation by the AKT family of protein kinases, which will require further investigation.

In summary, we provide here a dataset from small molecule, siRNA and cDNA screening that identified novel inhibitors and activators of cellular ATG4B activity and we share this data with the community for further investigations.

## Author Contributions

NP, KP, JC, CL, AA, and JF performed experiments and analyzed data. CG, AC, and DS provided material, expertise, and technical help. JK-V analyzed large datasets and provided bioinformatics expertise. NP and RK designed the study. RK wrote the manuscript and analyzed data. All authors read and contributed to the manuscript.

## Conflict of Interest Statement

The authors declare that the research was conducted in the absence of any commercial or financial relationships that could be construed as a potential conflict of interest.

## Footnotes

1 www.switchgeargenomics.com

2 www.ucl.ac.uk/chemibank

3 http://fiji.sc/Fiji

4 www.ucl.ac.uk/chemibank

## Supplementary Material

FIGURE S1 Sequence of the ATG4B promoter in the pLightSwitch vector (Switchgear Genomics, CA).

FIGURE S2 Small molecule screen for inhibitors and activators of cellular ATG4B acPvity. **(A)** HeLa cells stably expressing ATG4B (4B) displayed a strong activation of luciferase release into supernatants (SN) that was reduced in cells treated with Brefeldin A (BA). A *Z*′ factor for ATG4B + BA compared to ATG4B + DMSO was calculated for samples in a 384-well plate format and determined as 0.46. **(B)** Distribution of hits in the small molecule screen. Activators of ATG4B are shown on the left of the graph, and inhibitors on the right. **(C)** Distribution of raw value counts for each plate (triplicates of each plate, left panel) and values normalized to plate median (right panel). Over time, it was noted that the reporter cells showed an increase in basal luciferase release. We therefore decided to generate a new stable cell line after plate 44 that was more robust. Results displayed are from three independent replicates and statistical significance was determined using a two-sided paired *T*-Test (^∗∗∗^*p* < 0.001).

FIGURE S3 STK683963 does not activate the ATG4B-luciferase promoter construct. HEK293T cells were transfected with pLightSwitch-ATG4B luciferase and renilla luciferase activity was measured after 24 h. Biochanin A (Bioch) and Genistein (Genist) significantly up-regulater promoter-dependent luciferase, whereas STK683963 has no effect on ATG4B promoter activity. RLU, relative light unit. Results displayed are from three independent replicates and statistical significance was determined using a two-tailed paired *T*-Test (^∗∗^*p* < 0.01, ^∗^*p* < 0.05).

FIGURE S4 HeLa-ActinLC3dNGLUC cells were treated with the indicated concentrations of STK683964 overnight and cell viability was determined using the cell counting kit 8 (CCK8). No obvious effect on viability was observed at the indicated concentrations.

FIGURE S5 cDNA expression screen. **(A)** Heatmap of normalized luciferase values in the 384-well plate. The four 96-well plates of the cDNA human kinome library was pooled into one 384-well plate and 100 ng/well was transfected in HEK293T-ActinLC3dNGLUC cells. Positive (ATG4B transfection) and negative (untransfected) controls were included in the last two columns of the plate. **(B)** Distribution of samples relative to positive (red color) and negative controls (blue color). **(C,D)** The raw values were normalized to plate median and a B score calculation was applied. The distribution of positive and negative controls is shown in the left and right panel. The *Z*′ factor for this replicate was 0.41.

FILE S1 Information about the human kinase and human phosphatase siRNA libraries, including gene names and sequences for all siRNA oligonucleotides.

FILE S2 Information about the human kinase cDNA library.

FILE S3 R code used for statistical analysis of siRNA screening data.

FILE S4 Raw and normalized data from siRNA screening.

FILE S5 Raw and normalized data from cDNA overexpression screening.
