# Supplementary material for: Identification of Kinases and Phosphatases That Regulate ATG4B Activity by siRNA and Small Molecule Screening in Cells
Source: Front Cell Dev Biol. 2018 Nov 1;6:148. doi: 10.3389/fcell.2018.00148 (PMC6221980; doi:10.3389/fcell.2018.00148)
Supplement: FIGURE S3 — STK683963 does not activate the ATG4B-luciferase promoter construct. HEK293T cells were transfected with pLightSwitch-ATG4B luciferase and renilla luciferase activity was measured after 24 h. Biochanin A (Bioch) and Genistein (Genist) significantly up-regulater promoter-dependent luciferase, whereas STK683963 has no effect on ATG4B promoter activity. RLU, relative light unit. Results displayed are from three independent replicates and statistical significance was determined using a two-tailed paired T-Test (∗∗p < 0.01, ∗p < 0.05). [file Table_3.DOCX]

#read all files in a directory while deleting first 6 rows in all files

txt_files = list.files();

data = lapply(txt_files, read.table, skip = 6, header = TRUE, nrows= 768, sep = "\t")

#remove duplicates

uniquedata <- lapply(data, unique)

#transform in 96 wells

# add 1 2 3 4 row

replicateCol <- rep(c(1,2), times=96)

replicateRow <- rep(c(1,2), each=24, times=8)

dataReplicate <- lapply(uniquedata, cbind,"replicateRow"= replicateRow)

dataReplicate2 <- lapply(dataReplicate, cbind,"replicateCol"= replicateCol)

rep1 <- lapply(dataReplicate2, subset, replicateCol == 1 & replicateRow == 1)

rep2 <- lapply(dataReplicate2, subset, replicateCol == 1 & replicateRow == 2)

rep3 <- lapply(dataReplicate2, subset, replicateCol == 2 & replicateRow == 1)

rep4 <- lapply(dataReplicate2, subset, replicateCol == 2 & replicateRow == 2)

col96 <- read.table("/Users/robin/Documents/LAB/DATA/EnVIsion/screening_2013/96.txt")

colnames(col96) <- c("A1stuff")

rep1f <- lapply(rep1, cbind,"position"= col96$A1stuff)

rep2f <- lapply(rep2, cbind,"position"= col96$A1stuff)

rep3f <- lapply(rep3, cbind,"position"= col96$A1stuff)

rep4f <- lapply(rep4, cbind,"position"= col96$A1stuff)

rep1g <- lapply(rep1f, subset, select = c(Well,position,Result))

rep2g <- lapply(rep2f, subset, select = c(Well,position,Result))

rep3g <- lapply(rep3f, subset, select = c(Well,position,Result))

rep4g <- lapply(rep4f, subset, select = c(Well,position,Result))

#save all as text files

plates <- c("plate01","plate02","plate03","plate04", "plate05", "plate06", "plate07", "plate08", "plate09", "plate10", "plate11", "plate12", "plate13", "plate14", "plate15")

names(rep1g) <- plates

names(rep2g) <- plates

names(rep3g) <- plates

names(rep4g) <- plates

sapply(names(rep1g),

function (x) write.table(rep1g[[x]], file=paste(x, "rep1.txt"), col.names = FALSE, row.names = FALSE, quote = FALSE, sep="\t" ) )

sapply(names(rep2g),

function (x) write.table(rep2g[[x]], file=paste(x, "rep2.txt") , col.names = FALSE,row.names = FALSE, quote = FALSE, sep="\t" ) )

sapply(names(rep3g),

function (x) write.table(rep3g[[x]], file=paste(x, "rep3.txt") , col.names = FALSE, row.names = FALSE, quote = FALSE, sep="\t" ) )

sapply(names(rep4g),

function (x) write.table(rep4g[[x]], file=paste(x, "rep4.txt"), col.names = FALSE, row.names = FALSE, quote = FALSE, sep="\t" ) )

library(cellHTS2)

experimentName <- "siKinasePhosph"

dataPath <- "/Users/robin/Documents/LAB/DATA/EnVIsion/screening_2013/replicates96"

rev(dir(dataPath))

x <- readPlateList("Platelist.txt", name=experimentName, path=dataPath)

state(x)

x <- configure(x, descripFile="Description.txt", confFile="plateConf.txt", logFile="Screenlog.txt" ,path=dataPath)

state(x)

table(wellAnno(x))

xn <- normalizePlates(x, scale="multiplicative", log=FALSE, method="median")

xn <- annotate(xn, geneIDFile="Annotate.txt", path=dataPath)

xsc <- scoreReplicates(xn, sign="+", method="zscore")

xsc <- summarizeReplicates(xsc, summary="mean")

scores <- Data(xsc)

ylim <- quantile(scores, c(0.001, 0.999), na.rm=TRUE)

boxplot(scores ~ wellAnno(x), col="lightblue", outline=FALSE, ylim=ylim)

setSettings(list(plateList=list(reproducibility=list(include=TRUE, map=TRUE), intensities=list(include=TRUE, map=TRUE)), screenSummary=list(scores=list(range=c(-4, 8), map=TRUE))))

out <- writeReport(raw=x, normalized=xn, scored=xsc, outdir=dataPath, force=TRUE)

Analysis of plate results in R. First I extracted the replicates by creating virtual plates, and then analysed the entire dataset in cellHTS2 package.
