# Supplementary material for: Identification of Kinases and Phosphatases That Regulate ATG4B Activity by siRNA and Small Molecule Screening in Cells
Source: Front Cell Dev Biol. 2018 Nov 1;6:148. doi: 10.3389/fcell.2018.00148 (PMC6221980; doi:10.3389/fcell.2018.00148)
Supplement: FILE S1 — Information about the human kinase and human phosphatase siRNA libraries, including gene names and sequences for all siRNA oligonucleotides. [file Data_Sheet_1.PDF]

**PROMOTER SEQUENCE in pLightSwitch_Prom:**

```
AGGACCTGCGCTTCCAGAGCGTGCGGCTTCTGAAGCCTAGGCGCCGGCCGGATCGATCGC
GCGCAGGGCGGACCCAGGCGGCCGGGGTGGGGGCGGCTGCGCTGCCCGAGGCGCCCGGCC
CAGAGACGGCGGCCGGGCCAAGGTCACACAGCGCCCACGCCCGCTCCCCCGCGCCCCGGC
CGCCGAGGCCCGGCGCCCCCGCCCCCCGCGTTCGGGCCGGCCGCGGGGCCTCAGTTTCCC
CGCAGGGCCGGGTCGGGGCGGGGGCGTGGCGGCCCGGGGCCCGCGTACCTGTGGAAGGAG
ACGGCGCGCTTCTCGCCCTTTCCCTGCCGGTTGGAGCAGTTCACGGCCGCACAGCAGATC
ACCATCGCGGGCCTTGGCCCAGCCGCGCAGCCAGGCCCCGGCCCTAGCCGCCCGCCCGCC
CGCGGACCGCCCCGAGGGAGGGAGCGCGGCGGCGACACGGCTCGGGACGTGGGCCGGCCC
GCGGCGTCCGCGCCGTACGGCAAGATGGAGGCGCAGGCGCCCGCAGCGGGCCCGCCCCCG
CCCCAGCCCCCGCCCGCGTCCTCGCCAGCCGCGGGTTCGGGCGTCTTCGGACCAGCGGGG
CGCCGCAGGCCCCTCGCAGCGTCCGTCGGCAGGCGGGCAGACGGGCGGGGGAGTCGCCCC
GGCGGGGCAAGTCCGTACCGCGACATGGGCGCGCCGAGCACGTCCGTACCGCAAGATGGC
TGCTCGGACGGGGACAGAGCTCGCCTCTGCCGCCTCGACAACTGCTCCTGGGTCCTCTAA
GAGGAGGAAGCGCCACCCATGGCACACAGTGTCCCGTCGGACAGCAGAACCAGCCGTCGT
CCCACGACACGACCCCATGCCGCCCGCAGGGCGCCCCGGGGCTCGCGTCGGCCCGGCCGT
ACGCCAAAATGGCGGCTCCCGCGTATTTCCGCTCGCGCGCCGTATCGTCTTCGCCGCCTG
CGCCGGCACACCTATTGGCCCCCGCGGCGTCCCGTCGCCGCGTCGCGTTGCTGGCCCGTC
GGAGCGACGCCGCTCGGGTCAGTCGGCGGCCGGACTGGGAAGATGGACGCAGGTGAGGAG
TT
```

**Supplemental Figure 1:** Sequence of the ATG4B promoter in the pLightSwitch vector (Switchgear Genomics, CA).

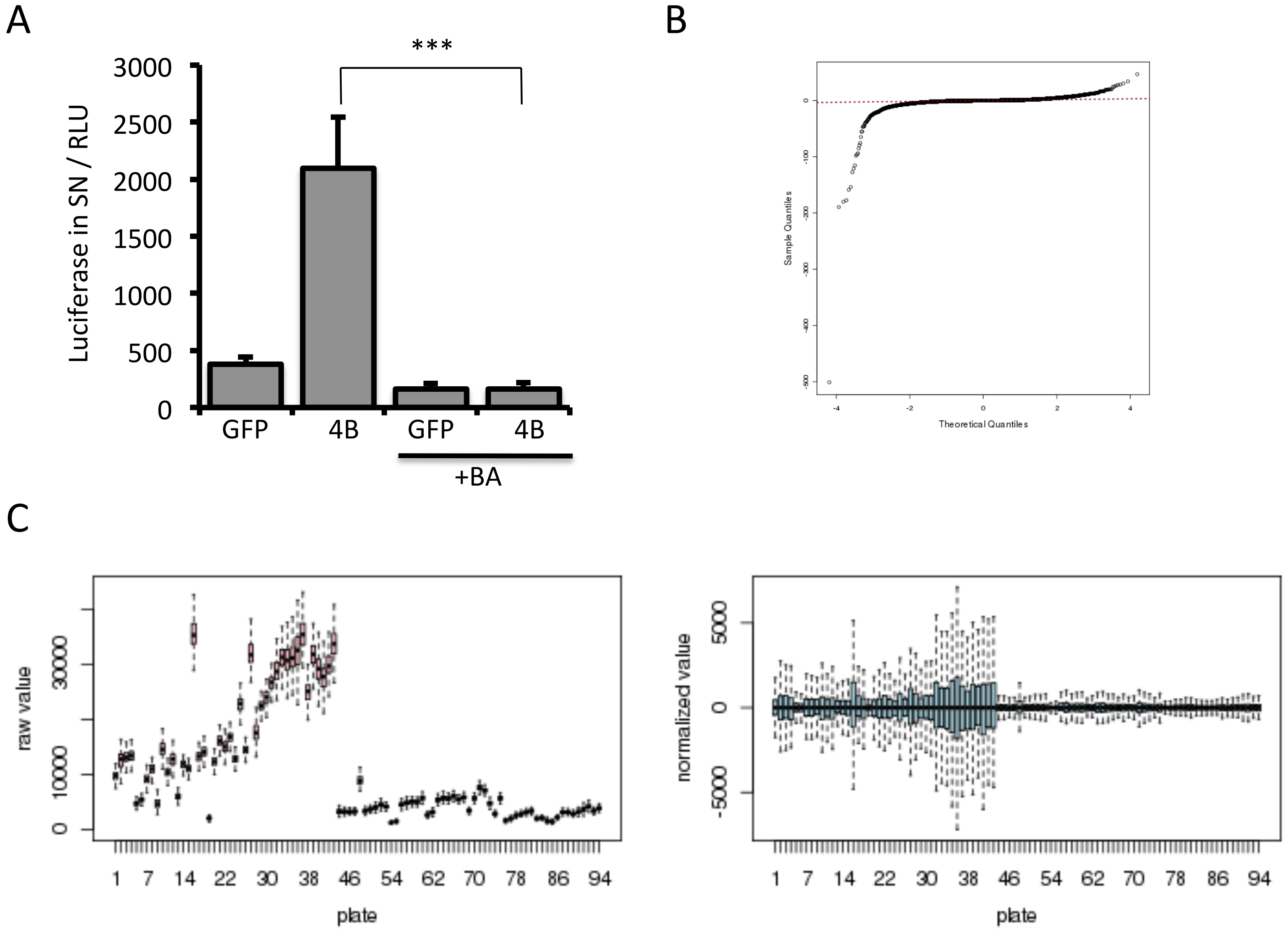


**Supplemental Figure 2:** Small molecule screen for inhibitors and activators of cellular ATG4B activity. A, HeLa cells stably expressing ATG4B (4B) displayed a strong activation of luciferase release into supernatants (SN) that was reduced in cells treated with Brefeldin A (BA). A Z' factor for ATG4B+BA compared to ATG4B+DMSO was calculated for samples in a 384-well plate format and determined as 0.46. B, Distribution of hits in the small molecule screen. Activators of ATG4B are shown on the left of the graph, and inhibitors on the right. C, Distribution of raw value counts for each plate (triplicates of each plate, left panel) and values normalised to plate median (right panel). Over time, it was noted that the reporter cells showed an increase in basal luciferase release. We therefore decided to generate a new stable cell line after plate 44 that was more robust. Results displayed are from three independent replicates and statistical significance was determined using a two-sided paired T-Test ($p<0.001$ = ***).

Delete?

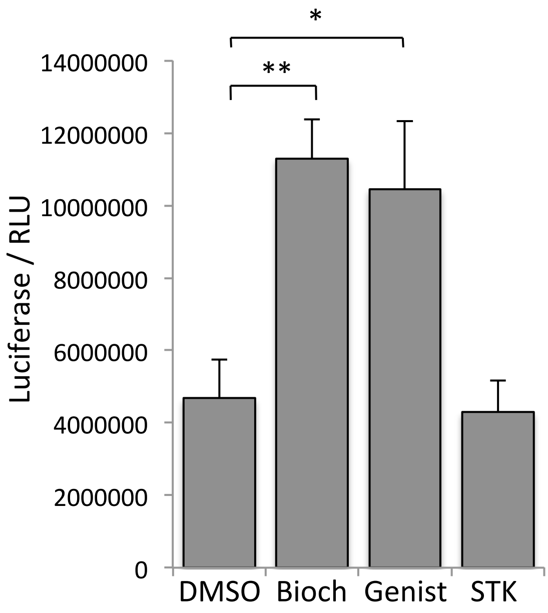


**Supplemental Figure 3:** STK683963 does not activate the ATG4B-luciferase promoter construct. HEK293T cells were transfected with pLightSwitch-ATG4Bluciferase and renilla luciferase activity was measured after 24 h. Biochanin A (Bioch) and Genistein (Genist) significantly up-regulater promoter-dependent luciferase, whereas STK683963 has no effect on ATG4B promoter activity. RLU, relative light unit. Results displayed are from three independent replicates and statistical significance was determined using a two-tailed paired T-Test ($p<0.01$ = **; $p<0.05$ = *).

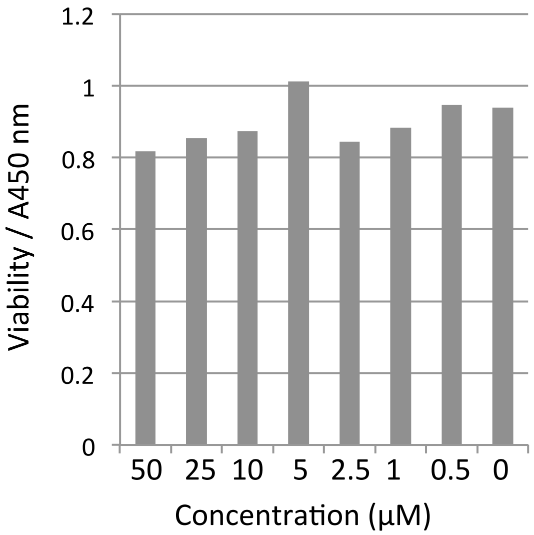


**Supplemental Figure 4:** HeLa-ActinLC3dNGLUC cells were treated with the indicated concentrations of STK683964 overnight and cell viability was determined using the cell counting kit 8 (CCK8). No obvious effect on viability was observed at the indicated concentrations.

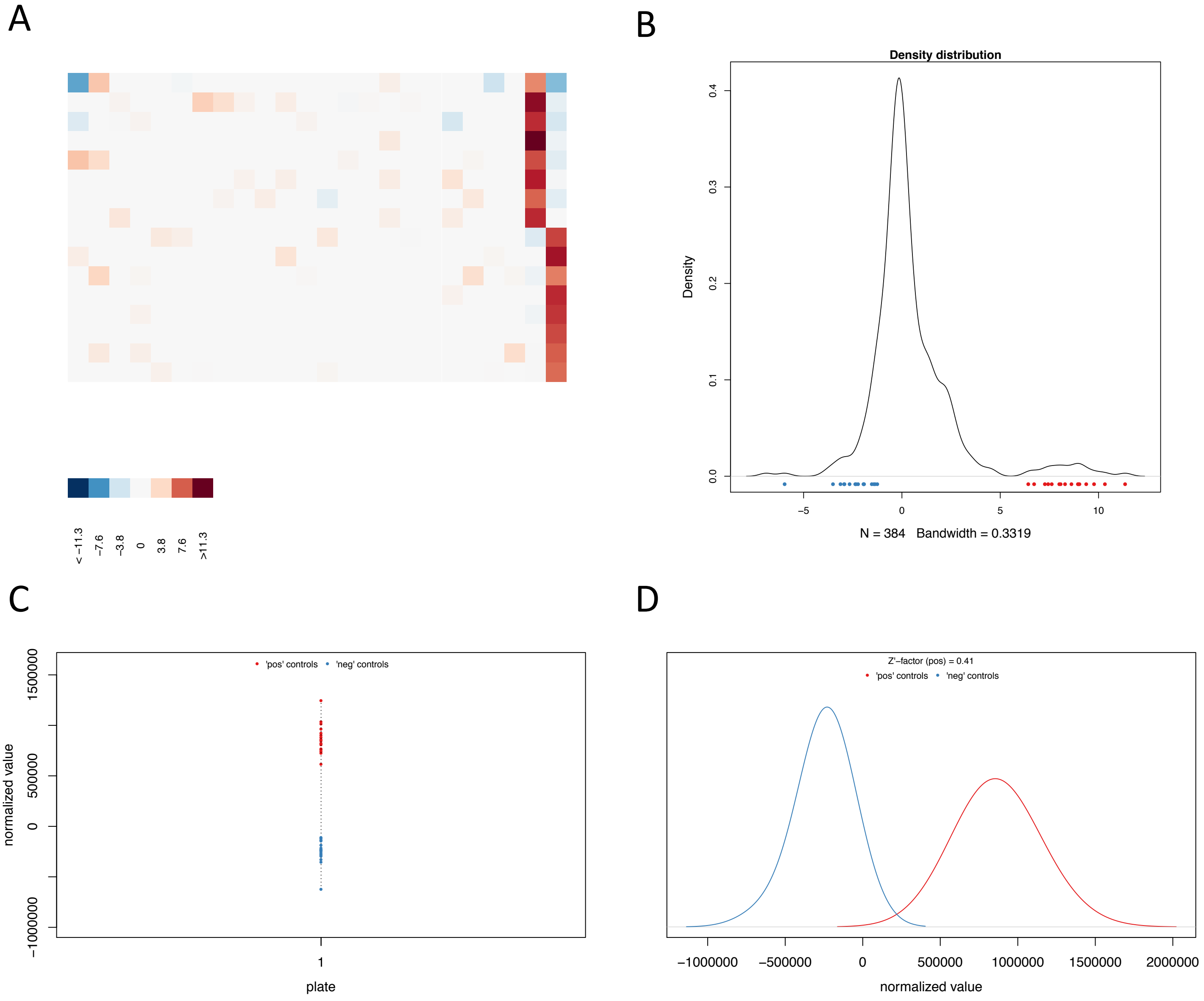


**Supplemental Figure 5:** cDNA expression screen. A, Heatmap of normalised luciferase values in the 384-well plate. The four 96-well plates of the cDNA human kinome library was pooled into one 384-well plate and 100 ng/well was transfected in HEK293T-ActinLC3dNGLUC cells. Positive (ATG4B transfection) and negative (untransfected) controls were included in the last two columns of the plate. B, Distribution of samples relative to positive (red colour) and negative controls (blue colour). C, D, The raw values were normalised to plate median and a B score calculation was applied. The distribution of positive and negative controls is shown in the left and right panel. The Z' factor for this replicate was 0.41.
